# Supplementary material for: Maternal polycystic ovary syndrome and Offspring’s Risk of Cardiovascular diseases in Childhood and Young Adulthood
Source: Nat Commun. 2024 Nov 29;15:10414. doi: 10.1038/s41467-024-54795-w (PMC11607077; doi:10.1038/s41467-024-54795-w)
Supplement: Supplementary file 1 — Supplementary Information [file 41467_2024_54795_MOESM1_ESM.pdf]

Supplementary Information

Maternal polycystic ovary syndrome and offspring's risk of cardiovascular diseases in childhood and young adulthood

Tables of Contents

Supplementary Table 1. Hazard ratios and 95% confidence intervals for overall cardiovascular disease in the offspring according to maternal polycystic ovary syndrome, by attained age
Supplementary Table 2. Sub-analyses of the association between maternal polycystic ovary syndrome and cardiovascular disease in offspring
Supplementary Table 3. Hazard ratios and 95% confidence intervals for overall and specific cardiovascular diseases in offspring according to maternal polycystic ovary syndrome in the propensity-score-matched sub-cohort
Supplementary Table 4. Description of the Danish and Swedish national registers used in our study
Supplementary Table 5. International Classification of Diseases codes for polycystic ovary syndrome and cardiovascular disease
Supplementary Table 6. Baseline characteristics of the propensity-score-matched sub-cohort
Supplementary Figure 1. Cumulative incidence of overall cardiovascular disease in offspring according to maternal polycystic ovary syndrome
Supplementary note 1. Description of the source, measurement, and categorization of covariates
Supplementary note 2. Description of the mediation analysis

Supplementary Table 1. Hazard ratios and 95% confidence intervals for overall cardiovascular disease in offspring according to maternal polycystic ovary syndrome, by attained age

Exposure	Number of events	Event rate, per 10,000 person-years	HR (95% CI)	
			Model 1 [a]	Model 2 [b]
**Attained age [c] <10 years (N=6 839 703)**				
No PCOS	56 445	8.98	1.0 (Reference)	1.0 (Reference)
PCOS	729	17.10	1.87 (1.74-2.01)	1.22 (1.13-1.31)
**10 ≤ Attained age ≤ 19 years (N=5 619 120)**				
No PCOS	74 881	15.71	1.0 (Reference)	1.0 (Reference)
PCOS	376	22.05	1.55 (1.40-1.71)	1.14 (1.03-1.26)
**20 ≤ Attained age ≤ 29 years (N=3 963 638)**				
No PCOS	121 440	38.78	1.0 (Reference)	1.0 (Reference)
PCOS	267	49.07	1.32 (1.17-1.48)	1.22 (1.08-1.37)
**30 ≤ Attained age ≤ 39 years (N=2 267 578)**				
No PCOS	104 457	68.72	1.0 (Reference)	1.0 (Reference)
PCOS	100	76.44	1.14 (0.94-1.39)	1.16 (0.95-1.41)
**Attained age ≥ 40 years (N=835 515)**				
No PCOS	25 559	86.56	1.0 (Reference)	1.0 (Reference)
PCOS	20	136.55	1.60 (1.04-2.48)	1.58 (1.02-2.46)

Abbreviations: PCOS, polycystic ovary syndrome; HR, hazard ratio; CI, confidence interval.
[a] Model 1 was unadjusted.
[b] Model 2 was adjusted for sex, country and calendar year of birth, maternal country of origin, parity, age, education, and marital status at the time of birth, hypertensive disorders, diabetes, and psychiatric disorders before or during the index pregnancy, and family history of cardiovascular disease.
[c] Attained age refers to the age of study participants from their entry into the cohort to their exit from the cohort (i.e., having a CVD diagnosis, death, emigration, or the latest date in our available datasets); i.e. attained age is equivalent to the age at the respective follow-up.

Supplementary Table 2. Sub-analyses of the association between maternal polycystic ovary syndrome and cardiovascular disease in offspring

Exposure	Number of events	Event rate, per 10 000 person-years	HR (95% CI)	
			Model 1[a]	Model 2[b]
**Stratified analysis according to offspring's sex**				
**Boy**				
No PCOS	189 274	23.03	1.0 (Reference)	1.0 (Reference)
PCOS	791	23.00	1.53 (1.44-1.65)	1.20 (1.11-1.28)
**Girl**				
No PCOS	193 508	24.89	1.0 (Reference)	1.0 (Reference)
PCOS	701	21.77	1.53 (1.43-1.65)	1.22 (1.13-1.32)
P-value for multiplicative interaction between PCOS and sex			0.007	0.008
**Stratified analysis according to offspring's country of birth**				
**Denmark**				
No PCOS	130 768	23.36	1.0 (Reference)	1.0 (Reference)
PCOS	451	20.88	1.50 (1.37-1.65)	1.20 (1.09-1.31)
**Sweden**				
No PCOS	252 014	24.22	1.0 (Reference)	1.0 (Reference)
PCOS	1041	23.14	1.55 (1.46-1.64)	1.21 (1.13-1.28)
P-value for multiplicative interaction between PCOS and country of birth			0.01	0.03
**Stratified analysis according to maternal ART**				
**Without ART**				
No PCOS	380 506	23.96	1.0 (Reference)	1.0 (Reference)
PCOS	1320	22.69	1.57 (1.48-1.65)	1.20 (1.14-1.27)
**With ART**				
No PCOS	2276	19.75	1.0 (Reference)	1.0 (Reference)
PCOS	172	20.44	1.09 (0.93-1.27)	1.06 (0.90-1.24)
P-value for multiplicative interaction between PCOS and ART			<0.0001	0.09
**Restricted to offspring with complete data on maternal smoking (N=4 617 257)** [c]				
No PCOS	163 096	19.47	1.0 (Reference)	1.0 (Reference)
PCOS	1187	21.00	1.43 (1.35-1.51)	1.20 (1.14-1.27)
**Restricted to offspring with complete data on maternal body-mass index (N=3 565 133)** [d]				
No PCOS	273 507	25.95	1.0 (Reference)	1.0 (Reference)
PCOS	595	25.43	1.57 (1.45-1.70)	1.25 (1.16-1.36)
**Restricted to offspring born in Denmark since 1995 and in Sweden since 2001 (N=2 765 917)**				
No PCOS	49 500	15.51	1.0 (Reference)	1.0 (Reference)
PCOS	798	19.00	1.27 (1.18-1.36)	1.17 (1.09-1.25)
**Restricted to CVD cases identified from national patient registers**				
No PCOS	381 542	23.32	1.0 (Reference)	1.0 (Reference)
PCOS	1488	21.39	1.54 (1.46-1.62)	1.20 (1.14-1.27)
**Year of PCOS diagnosis** [e]				

No PCOS	382 782	23.93	1.0 (Reference)	1.0 (Reference)
<1990	196	30.15	1.17 (1.02-1.35)	1.21 (1.05-1.39)
1990-2003	423	23.02	1.56 (1.42-1.71)	1.23 (1.12-1.35)
>2003	873	20.92	1.75 (1.63-1.87)	1.20 (1.12-1.28)
**Imputed data by multiple imputation by chained equations [f]**				
No PCOS	382 782	23.93	1.0 (Reference)	1.0 (Reference)
PCOS	1492	22.41	1.59 (1.52-1.66)	1.20 (1.15-1.26)

Abbreviations: PCOS, polycystic ovary syndrome; ART, assisted reproductive treatment; HR, hazard ratio; CI, confidence interval.

[a] Model 1 was unadjusted.
[b] Model 2 was adjusted for sex, country and calendar year of birth, maternal origin of country, parity, age, education, and marital status at the time of birth, hypertensive disorders, diabetes, and psychiatric disorders before or during the index pregnancy, and family history of cardiovascular diseases.
[c] We further adjusted for maternal smoking in early pregnancy in addition to factors included in Model 2.
[d] We further adjusted for maternal body-mass index in early pregnancy in addition to factors included in Model 2.
[e] There were 2183 women who had a diagnosis of polycystic ovary syndrome before 1990, 111 23 between 1990 and 2003, and 38 417 after 2003.
[f] We imputed missing variables using multiple imputation by the chained equations method to generate five imputations. We used logistic regression to impute the following categorical variables: offspring’s sex, maternal origin of country, education, and marital status.

Supplementary Table 3. Hazard ratios and 95% confidence intervals for overall and specific cardiovascular diseases in offspring according to maternal polycystic ovary syndrome in the propensity score matched sub-cohort

Exposure	Number of events	Rate, per 10,000 person-years	Model 1[a] HR (95% CI)	Model 2[b] HR (95% CI)
**Overall CVD**				
No PCOS	2627	23.74	1.0 (Reference)	1.0 (Reference)
PCOS	1492	22.41	1.38 (1.29-1.48)	1.19 (1.10-1.27)
**Ischemic heart disease**				
No PCOS	47	0.42	1.0 (Reference)	1.0 (Reference)
PCOS	20	0.30	1.80 (1.03-3.15)	1.76 (0.98-3.15)
**Acute myocardial infarction**				
No PCOS	18	0.16	1.0 (Reference)	1.0 (Reference)
PCOS	8	0.12	2.36 (0.98-5.71)	2.51 (1.03-6.16)
**Stroke**				
No PCOS	92	0.81	1.0 (Reference)	1.0 (Reference)
PCOS	44	0.65	1.21 (0.82-1.78)	1.05 (0.70-1.58)
**Haemorrhagic stroke**				
No PCOS	25	0.22	1.0 (Reference)	1.0 (Reference)
PCOS	13	0.19	1.12 (0.55-2.27)	1.02 (0.48-2.17)
**Ischemic stroke**				
No PCOS	54	0.48	1.0 (Reference)	1.0 (Reference)
PCOS	29	0.43	1.46 (0.89-2.38)	1.16 (0.39-1.95)
**Heart failure**				
No PCOS	38	0.34	1.0 (Reference)	1.0 (Reference)
PCOS	12	0.18	0.66 (0.33-1.31)	0.72 (0.35-1.49)
**Atrial fibrillation**				
No PCOS	62	0.55	1.0 (Reference)	1.0 (Reference)
PCOS	18	0.27	0.99 (0.58-1.72)	1.18 (0.68-2.05)
**Hypertensive disorders**				
No PCOS	349	3.09	1.0 (Reference)	1.0 (Reference)
PCOS	146	2.16	1.54 (1.26-1.89)	1.38 (1.11-1.71)
**Peripheral arterial disease**				
No PCOS	10	0.09	1.0 (Reference)	1.0 (Reference)
PCOS	6	0.09	1.46 (0.50-4.27)	1.77 (0.58-5.38)

Abbreviations: PCOS, polycystic ovary syndrome; CVD, cardiovascular disease; HR, hazard ratio; CI, confidence interval.

[a] Model 1 was unadjusted.

[b] Model 2 was adjusted for sex, country and calendar year of birth, and maternal age at the time of birth.

Supplementary Table 4. Description of the Danish and Swedish national registers used in our study

Data source	Information retrieved from the register	Period covered
**The Danish registers**		
Danish Civil Registration System	Sex, birth date, place of birth, vital status, marital status, emigration and immigration, and linkage to relatives	1968-2016
Danish Integrated Database for Labour Market Research	Mother's education level	1980-2016
Danish Medical Birth Register	Birth date, sex, gestational age, birth weight, singleton status, mother's height, weight, and smoking in early pregnancy, age at delivery and parity	1973-2016
Danish National Patient Registry	Information on inpatient and outpatient care (diagnosis, date)	Inpatient care: 1977-2016; specialized outpatient care and emergency department contacts: 1995-2016
Danish Register of Causes of Death	Date of death and cause of death	1970-2016
**The Swedish registers**		
Swedish Total Population Register	Sex, birth date, place of birth, marital status, migration, and civil status	1968-2014
Swedish Multi-Generation Register	Relationships for all residents born since 1932 [a]	1961-2014
Swedish Register of Education	Mother's education	1985-2014
Swedish Medical Birth Register	Birth date, sex, gestational age, birth weight, singleton status, mother's height, weight, and smoking status in early pregnancy, age at delivery and parity, and complications during pregnancy	1973-2014
Swedish Patient Register	Information on inpatient and outpatient care (diagnosis, date)	Inpatient care: 1964-2020 (its coverage became nationwide since 1987); specialized outpatient care: 2001-2020
Swedish Cause of Death Register	Date and cause of death	1952-2020

[a] The index persons must have been registered since 1961 in order to be included.

Supplementary Table 5. International Classification of Diseases codes for polycystic ovary syndrome and cardiovascular diseases

	ICD-8	ICD-9	ICD-10
**Polycystic ovary syndrome**			
Denmark	25690		E282
Sweden	25690	256E	E282
**Overall cardiovascular disease**			
Denmark	390-458		I00-I99
Sweden	390-458	390-459	I00-I99
**Ischemic heart disease**			
Denmark	410-414		I20-I25
Sweden	410-414	410-414	I20-I25
**Acute myocardial infarction**			
Denmark	410		I21, I22
Sweden	410	410	I21, I22
**Stroke**			
Denmark	430, 431, 433, 434, 436		I60, I61, I63, I64
Sweden	430, 431, 433, 434, 436	430, 431, 433, 434, 436	I60, I61, I63, I64
**Haemorrhagic stroke (except subarachnoid haemorrhage)**			
Denmark	431		I61
Sweden	431	431	I61
**Ischemic stroke**			
Denmark	433, 434		I63, I64
Sweden	433, 434	433, 434	I63, I64
**Heart failure**			
Denmark	42709, 42710, 42711, 42719		I110, I130, I132, I50
Sweden	427.00, 427.10	428	I11.0, I13.0, I13.2, I50
**Atrial fibrillation/flutter**			
Denmark	42793, 42794		I48
Sweden	427.92	427D	I48
**Hypertensive disease**			
Denmark	400-404		I10-I15, O10, O11
Sweden	400-404	401-405, 642A, 642B, 642C, 642H	I10-I15, O10, O11
**Peripheral arterial disease**			
Denmark	440, 44399, 445		I70, I739
Sweden	440, 4438, 4439, 445	440, 443W, 443X	I70, I73.9

Abbreviation: ICD, International Statistical Classification of Diseases and Related Health Problems.

Polycystic ovary syndrome diagnoses were extracted from the Danish and Swedish patient registers and the Swedish Medical Birth Register. Overall cardiovascular disease was identified using primary or secondary diagnoses in the national patient registers or from the underlying cause of death in the cause of death register. Ischemic heart disease, stroke, and heart failure were identified based on the primary diagnosis in the national patient registers or from the underlying cause of death in the cause of death register. Hypertensive disease, atrial fibrillation and peripheral artery disease were identified based on the primary or secondary diagnoses in national patient registers.

Supplementary Table 6. Baseline characteristics of the propensity-score-matched sub-cohort

Characteristics	Exposure	
	No maternal PCOS (N=51 723)	Maternal PCOS (N=51 723)
	N (%)	N (%)
**Characteristics of the offspring**		
**Study country**		
Denmark	19 516 (37.3)	20 235 (39.1)
Sweden	32 207 (62.3)	31 488 (60.9)
**Calendar year of birth**		
1973-1978	5252 (10.2)	523 (1.0)
1979-1984	5631 (10.9)	1088 (2.1)
1985-1990	6811 (13.2)	2054 (4.0)
1991-1996	7540 (14.6)	3971 (7.7)
1997-2002	7137 (13.8)	6428 (12.4)
2003-2008	8493 (16.4)	13 267 (25.7)
2009-2016	10 859 (21.0)	24 392 (47.2)
**Sex**		
Boy	26 622 (51.5)	26 683 (51.6)
Girl	25 094 (48.5)	25 040 (48.4)
Unknown	7 (0.01)	0
**Congenital heart disease**		
No	50 890 (98.4)	50 480 (97.6)
Yes	833 (1.6)	1243 (2.4)
**Preterm birth**		
No	46 336 (89.6)	47 536 (91.9)
Yes	2957 (5.7)	3683 (7.1)
Unknown	2430 (4.7)	504 (1.0)
**Small for gestational age** [a]		
No	46 410 (89.7)	47 419 (91.7)
Yes	5313 (10.3)	4304 (8.3)
**Large for gestational age** [b]		
No	46 893 (90.7)	44 900 (86.8)
Yes	4830 (9.3)	6823 (13.2)
**Maternal characteristics**		
**Country of origin same as the study country**		
No	10 503 (20.3)	10 550 (20.4)
Yes	41 217 (79.7)	41 170 (79.6)
Unknown	3 (0.01)	3 (0.01)
**Age (years) at the time of the index birth**		
≤19	1672 (3.2)	1488 (2.9)
20-24	10 689 (20.7)	9699 (18.8)
25-29	18 333 (35.4)	17 895 (34.6)
30-34	14 149 (27.4)	15 671 (30.3)
≥35	6880 (13.3)	6970 (13.5)
**Level of education at the time of the index birth**		
Primary and lower secondary	9820 (19.0)	9931 (19.2)
Upper secondary	22 046 (42.6)	22 076 (42.7)
Bachelor or higher	19 390 (37.5)	19 240 (37.2)
Unknown	467 (0.9)	476 (0.9)

**Marital status at the time of the index birth**		
Not married/no registered partnership	26 499 (51.2)	26 554 (51.3)
Married/registered partnership	24 858 (48.1)	24 860 (48.1)
Unknown	366 (0.7)	319 (0.6)
**Parity at the time of the index birth**		
1	28 826 (55.7)	28 453 (55.0)
2	16 931 (32.7)	16 924 (32.7)
≥3	5966 (11.5)	6346 (12.3)
**Smoking in early pregnancy** [c]		
No	31 893 (79.0)	41 577 (83.9)
Yes	6175 (15.3)	5953 (12.0)
Unknown	2297 (5.7)	2020 (4.1)
**Body-mass index in early pregnancy (kg/m^2)** [d]		
<18.5	1107 (3.2)	739 (1.64)
18.5-24.9	17 016 (48.9)	16 424 (36.4)
25.0-29.9	6819 (19.6)	11 850 (26.3)
≥30.0	3222 (9.3)	11 264 (25.0)
Unknown	6615 (19.0)	4806 (10.7)
**Assisted reproductive treatment during index pregnancy** [e]		
No	28 831 (96.5)	38 028 (82.9)
Yes	1036 (3.5)	7869 (17.1)
**Diabetes before or during index pregnancy**		
No	48 787 (94.3)	48 460 (93.7)
Yes	2936 (5.7)	3263 (6.3)
**Hypertensive disorders before or during index pregnancy**		
No	47 445 (91.7)	47 779 (92.4)
Yes	4278 (8.3)	3944 (7.6)
**Psychiatric disorders before or during index pregnancy**		
No	45 764 (88.5)	45 804 (88.6)
Yes	5959 (12.5)	5919 (11.4)
**Family history of cardiovascular disease before the index birth**		
No	29 157 (56.4)	28 676 (55.4)
Yes	22 566 (43.6)	23 047 (44.6)

Abbreviation: PCOS, polycystic ovary syndrome.

[a] Small for gestational age, birth weight below the 10th percentile of the sex-specific standard curve for normal fetal growth.

[b] Large for gestational age, birth weight above the 10th percentile of the sex-specific standard curve for normal fetal growth.

[c] Maternal smoking was available since 1982 in Sweden and since 1991 in Denmark.

[d] Maternal body-mass index has been available since 1982 in Sweden and since 2003 in Denmark.

[e] Maternal assisted reproductive treatment has been available since 1994 in Denmark and since 1995 in Sweden.

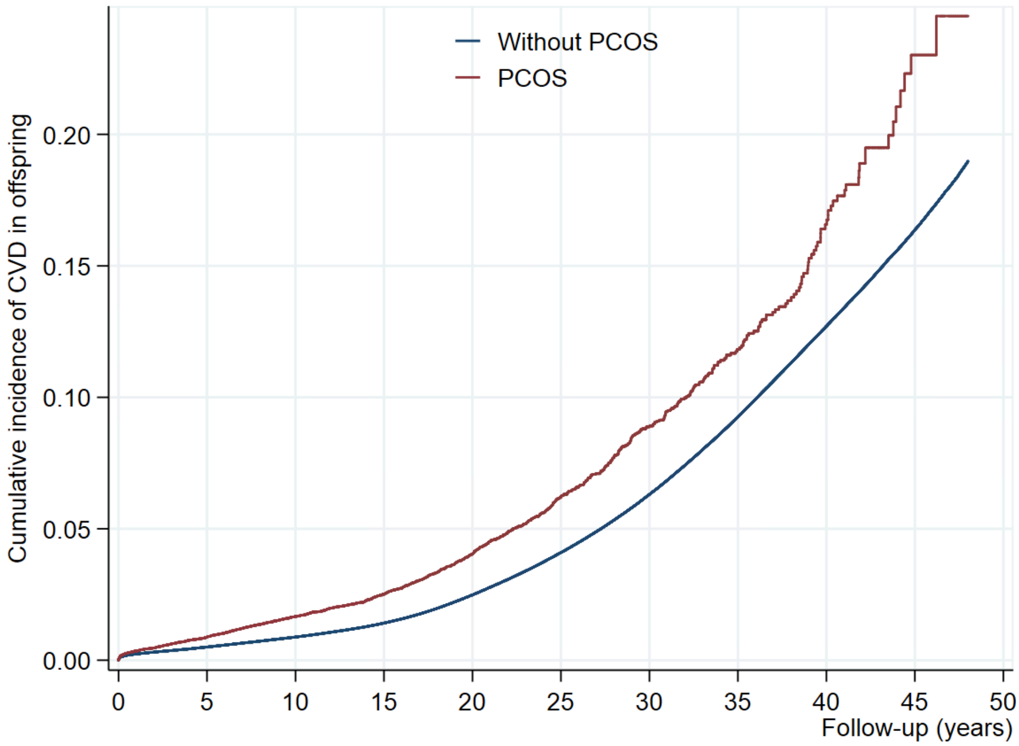

Number at risk

	0	5	10	15	20	25	30	35	40	45	50
Without PCOS	6787980	6363379	5590521	4756548	3954689	3138761	2265012	1497509	835187	236424	0
PCOS	51723	44595	28605	16101	9182	5217	2571	1200	480	98	0

Supplementary Figure 1. Cumulative incidence of overall cardiovascular disease in offspring according to maternal polycystic ovary syndrome

PCOS, polycystic ovary syndrome.

Supplementary note 1. Description of the source, measurement, and categorization of covariates

1. Offspring characteristics

Sex (boy or girl), calendar year of birth (1973-1978, 1979-1984, 1985-1990, 1991-1996, 1997-2002, 2003-2008, or 2009-2016), gestational age, and birth weight were obtained from the Danish and Swedish Medical Birth Registers. Preterm birth (yes/no) was defined as gestational age < 37 weeks at birth. Birth weight for gestational age was calculated according to the Scandinavian sex-specific reference curve for normal foetal growth, and further categorized into small for gestational age (<10th percentile), appropriate for gestational age (between the 10th and 90th percentile), and large for gestational age (>90th percentile). Diagnoses of congenital heart disease were obtained from the Danish National Patient Register (DNPR) and the Swedish Patient Register (SPR), using the following *International Statistical Classification of Diseases and Related Health Problems* (ICD) codes: 1) in Denmark: ICD-8: 746, 747; ICD-10: Q20-Q27; and 2) in Sweden: ICD-8: 746, 747; ICD-9:745, 746, 747; ICD-10: Q20-Q27. Diagnoses of diabetes were obtained from the DNPR, and SPR using the ICD codes: 1) in Denmark: ICD-8: 250 and ICD-10: E10-E14; and 2) in Sweden: ICD-8: ICD-8/9: 250 and ICD-10: E10-E14.

2. Maternal characteristics

Information on maternal country of origin (same as the study country or not) and marital status (married/registered partnership versus not) was obtained from the Danish Civil Registration System and the Swedish Total Population Register. Maternal educational level (primary and lower secondary, upper secondary, bachelor, or higher) was extracted from the Danish Integrated Database for Labour Market Research and the Swedish Register of Education.

From the Danish and Swedish Medical Birth Registers, we extracted information on maternal age (≤19, 20-24, 25-29, 30-34, or ≥35 years), parity (1, 2, ≥3) and assisted reproductive treatment (available since 1994 in Denmark and since 1995 in Sweden) at the time of birth, smoking (available since 1991 in Denmark and since 1982 in Sweden), and body-mass index

(available since 2003 in Denmark and since 1982 in Sweden) in early pregnancy. Body-mass index was further classified as underweight (<18.5 kg/m^2), normal weight (18.5–24.9 kg/m^2), overweight (25.0–29.9 kg/m^2), and obesity (≥30 kg/m^2).

Maternal comorbidities before or during the index pregnancy, including diabetes, hypertensive disease, and psychiatric disorders, were retrieved from the DNPR and the Swedish Medical Birth Register. Maternal diabetes was identified using the following ICD codes: 1) in Denmark: ICD-8: 249, 250; ICD-10: E10-E14, O24; and 2) in Sweden: ICD-8: 250; ICD-9: 250, 648A, 648W; ICD-10: E10-E14, O24. Maternal hypertensive disease was identified using the following ICD codes: 1) in Denmark: ICD-8: 40009, 40019, 40029, 40039, 40099, 40199, 63700, 63703, 63704, 63709, 63719, 76029; ICD-10: I10, I11, I12, I13, I15, O10, O11, O13, O14, O15, O16; and 2) in Sweden: ICD-8: 400, 401, 402, 403, 404, 63701, 63703, 63704, 63709, 63799, 63710; ICD-9: 401, 402, 403, 404, 405, 642A, 642B, 642C, 642D, 642E, 642F, 642G, 642H, 642X; ICD-10: I10, I11, I12, I13, I15, O10, O11, O13, O14, O15, O16. Maternal psychiatric disorders were identified using the following ICD codes: 1) Denmark: ICD-8: 290-315; ICD-10: F00-F99; and 2) in Sweden: ICD-8: 290-315; ICD-9: 290-319; and ICD-10: F00-F99.

Maternal family history of cardiovascular diseases was defined as a history of cardiovascular diseases among first-degree relatives. A mother's family relatives were identified through the Danish Civil Registration System and the Swedish Multi-Generation Register. We then extracted information on maternal relatives' cardiovascular disease diagnoses (ICD-8/9: 390–459, ICD-10: I00-I99) from the DNPR and SPR.

Supplementary note 2. Description of the mediation analysis

We explored the role of several potential mediators, i.e. preterm birth, small and large for gestational age (SGA and LGA), congenital heart disease, and diabetes in the association between maternal polycystic ovary syndrome (PCOS, the exposure of interest) and cardiovascular diseases (CVD, the outcome of interest) among offspring.

We performed the mediation analysis based on the counterfactual framework, which has been extensively described elsewhere.[1] We obtained the total effect of PCOS on the risk of CVD in offspring by comparing CVD risk among offspring exposed to maternal PCOS with that of unexposed offspring, broken down into direct and mediated effects. The direct effect (DE) represents the effect of maternal PCOS on CVD that was independent of preterm birth, SGA or LGA birth, and congenital heart disease. The mediated effect (ME) reflected the influence of maternal PCOS that could be explained by its influence on mediators. The proportion mediated by each mediator was then estimated as: [DE × (ME -1) / (DE × ME) -1] ×100%, where 0% reflects no mediated effect and 100% reflects no direct effect. We employed the SAS macro *%mediation* developed by Linda Valeri and Tyler Vanderweele[2] to perform the mediation analysis.

We modelled the exposure-mediator (*i.e.*, maternal PCOS-preterm birth, maternal PCOS-SGA birth, maternal PCOS-LGA birth, maternal PCOS- congenital heart disease, and maternal PCOS-diabetes) using logistic regression. The exposure-outcome (*i.e.*, maternal PCOS-offspring CVD) association was modelled using Cox regression, with an interaction term between the exposure and the mediator. We included the following covariates in the models: offspring's sex, country and year of birth, and maternal country of origin, parity, age, education, and marital status at the time of birth, BMI and smoking in early pregnancy, family history of CVD, hypertensive disease, diabetes, and psychiatric disorders before or during the index pregnancy.

As noted elsewhere,[3] the mediation analysis required strong assumptions: 1) no unmeasured exposure-outcome confounding, 2) no unmeasured exposure-mediator confounding, 3) no

unmeasured mediator-outcome confounding, and 4) none of the mediator-outcome confounders being affected by the exposure. Our observational data cannot rule out the possibility of unmeasured confounders, and the assumption of “no unmeasured mediator-outcome confounders” was especially likely to be violated. Therefore, we further examined the impact of unmeasured mediator-outcome confounding by using the sensitivity analysis technique.[4] Using congenital heart disease as an example, we first assumed that the effect of a strong unmeasured confounder *U* on CVD on the hazard ratio scale was 1.8, that the prevalence of *U* among offspring with congenital heart disease who were exposed to maternal PCOS was 40%, and that the prevalence of *U* among unexposed offspring with congenital heart disease was 45%. After introducing the *U* into the sensitivity analysis, we found that the direct effect was slightly increased (hazard ratio: 1.21, 95% confidence interval: 1.14-1.27), but the mediated effect was reduced and close to null (0.99, 0.98-0.99). We found similar patterns when we performed sensitivity analyses in case of diabetes, preterm birth and SGA or LGA birth.

Supplementary References

1. Robins JM, Greenland SJE. Identifiability and exchangeability for direct and indirect effects. *Epidemiology.* 1992:143-155.
2. Valeri L, VanderWeele TJ. Mediation analysis allowing for exposure–mediator interactions and causal interpretation: theoretical assumptions and implementation with SAS and SPSS macros. *Psychological methods.* 2013;18(2):137.
3. Lange T, Hansen JVJE. Direct and indirect effects in a survival context. *Epidemiology.* 2011:575-581.
4. VanderWeele TJJEjoe. Unmeasured confounding and hazard scales: sensitivity analysis for total, direct, and indirect effects. *European journal of epidemiology.* 2013;28(2):113-117.
