# Supplementary material for: Maternal polycystic ovary syndrome and Offspring’s Risk of Cardiovascular diseases in Childhood and Young Adulthood
Source: Nat Commun. 2024 Nov 29;15:10414. doi: 10.1038/s41467-024-54795-w (PMC11607077; doi:10.1038/s41467-024-54795-w)
Supplement: Supplementary file 2 — Reporting summary [file 41467_2024_54795_MOESM2_ESM.pdf]

nature portfolio

Corresponding author(s): Fen Yang

Last updated by author(s): Nov 6, 2024

Reporting Summary

Nature Portfolio wishes to improve the reproducibility of the work that we publish. This form provides structure for consistency and transparency in reporting. For further information on Nature Portfolio policies, see our Editorial Policies and the Editorial Policy Checklist.

Statistics

For all statistical analyses, confirm that the following items are present in the figure legend, table legend, main text, or Methods section.

n/a	Confirmed	
☐	☒	The exact sample size (*n*) for each experimental group/condition, given as a discrete number and unit of measurement
☐	☒	A statement on whether measurements were taken from distinct samples or whether the same sample was measured repeatedly
☐	☒	The statistical test(s) used AND whether they are one- or two-sided<br>*Only common tests should be described solely by name; describe more complex techniques in the Methods section.*
☐	☒	A description of all covariates tested
☐	☒	A description of any assumptions or corrections, such as tests of normality and adjustment for multiple comparisons
☐	☒	A full description of the statistical parameters including central tendency (e.g. means) or other basic estimates (e.g. regression coefficient) AND variation (e.g. standard deviation) or associated estimates of uncertainty (e.g. confidence intervals)
☐	☒	For null hypothesis testing, the test statistic (e.g. *F*, *t*, *r*) with confidence intervals, effect sizes, degrees of freedom and *P* value noted<br>*Give P values as exact values whenever suitable.*
☒	☐	For Bayesian analysis, information on the choice of priors and Markov chain Monte Carlo settings
☐	☒	For hierarchical and complex designs, identification of the appropriate level for tests and full reporting of outcomes
☒	☐	Estimates of effect sizes (e.g. Cohen's *d*, Pearson's *r*), indicating how they were calculated

Our web collection on statistics for biologists contains articles on many of the points above.

Software and code

Policy information about availability of computer code

Data collection	All data on study participants in our cohort were collected by Danish and Swedish national register holders. Following ethical approval, researchers obtained access to pseudonymized data; data were securely stored on Statistics Denmark's platform, accessible only to authorized researchers.
Data analysis	All data analyses for this study were conducted using SAS, version 9.4 (SAS Institute Inc), and RStudio, version 1.2.1578 (RStudio Inc).<br>Code availability: All data analyses for this study were conducted on a secure server at Statistics Denmark. We are not allowed to export files from this server, neither data nor statistical codes.

For manuscripts utilizing custom algorithms or software that are central to the research but not yet described in published literature, software must be made available to editors and reviewers. We strongly encourage code deposition in a community repository (e.g. GitHub). See the Nature Portfolio guidelines for submitting code & software for further information.

Data

Policy information about availability of data

All manuscripts must include a data availability statement. This statement should provide the following information, where applicable:

- Accession codes, unique identifiers, or web links for publicly available datasets
- A description of any restrictions on data availability
- For clinical datasets or third party data, please ensure that the statement adheres to our policy

The raw Danish and Swedish cohort data were collected by national register holders in each country and cannot be shared publicly due to restrictions in our ethical approval and data privacy laws. The cohort data generated in this study have been securely stored on a secure server at Statistics Denmark (https://www.dst.dk/), which also prohibits data sharing with external users. Researchers who meet legal requirements may apply for similar data from the relevant registers, provided they have obtained the necessary ethical approvals. The holders of the registers used in this study and their websites are: Statistics Denmark (https://www.dst.dk/), Statistics Sweden (https://www.scb.se/en/), and the National Board of Health and Welfare in Sweden (https://www.socialstyrelsen.se/en/). Processing times for data applications to register holders, once ethical approvals are granted, vary depending on demand and the capacity of each register holder and may range from several months to over a year. The time periods for the availability of the granted data are regulated by agreements between register holders and the respective researchers. Statistics Denmark has strict access restrictions and Danish data cannot be exported or stored outside Statistics Denmark's server.

Research involving human participants, their data, or biological material

Policy information about studies with human participants or human data. See also policy information about sex, gender (identity/presentation), and sexual orientation and race, ethnicity and racism.

Reporting on sex and gender	We used "sex" in our study only from a biological perspective. Sex (boy or girl) was obtained from the Danish and Swedish Medical Birth Registers. We presented results for all study participants included in our study.<br><br>A total of 3,514,004 (51.4%) boys and 3,324,391 (48.6%) girls were included in our cohort. We performed stratified analyses according to the offspring's sex and tested its interactions with maternal polycystic ovary syndrome and the offspring's cardiovascular diseases risk.
Reporting on race, ethnicity, or other socially relevant groupings	We extracted information on the mother's country of origin (same as the study country or not) and marital status (married/registered partnership versus not) from the Danish Civil Registration System and the Swedish Total Population Register, and the mother's educational level (primary and lower secondary, upper secondary, bachelor, or higher) from the Danish Integrated Database for Labour Market Research and the Swedish Register of Education. We considered these three socially relevant covariates confounders and adjusted for them in our multivariable model.<br><br>We did not report race or ethnicity in our study, as such information may not be collected in Nordic registers.
Population characteristics	Among 6,839,703 singletons included in this cohort, 51,723 (0.76%) singletons were born to mothers with polycystic ovary syndrome. Compared to their unexposed counterparts, offspring of mothers with polycystic ovary syndrome were more likely to be born after 2003, with a preterm birth or large for gestational at birth. Their mothers were more likely to be foreign-born, to have higher educational attainment, to be single, non-smoking, nulliparous, and obese, to have undergone assisted reproductive treatment, and to have had a diagnosis of diabetes, hypertensive disease, or psychiatric disorders before childbirth.
Recruitment	We included all live singleton births registered in the Danish and Swedish Medical Birth Registers during 1973-2016 and 1973-2014, respectively. The likelihood of selection bias was minimal.
Ethics oversight	The study was approved by the Danish Data Protection Agency (No. 2013-41-2569) and the Research Ethics Committee at Karolinska Institute in Stockholm (No. 2016/288-31/1 and 2021-03315). The boards do not request informed consent for register-based studies.

Note that full information on the approval of the study protocol must also be provided in the manuscript.

Field-specific reporting

Please select the one below that is the best fit for your research. If you are not sure, read the appropriate sections before making your selection.

☒ Life sciences ☐ Behavioural & social sciences ☐ Ecological, evolutionary & environmental sciences

For a reference copy of the document with all sections, see nature.com/documents/nr-reporting-summary-flat.pdf

Life sciences study design

All studies must disclose on these points even when the disclosure is negative.

Sample size	We included individuals born in Denmark and Sweden from 1973 to 2016 and 1973 to 2014, respectively. Taking stroke as outcome; its incidence rate is 0.15% in Denmark and Sweden, while the estimated exposure (maternal policystic ovary syndrome) prevalence was 0.6%. With these rates, aiming for a statistical power of 80%, a significance level (α) of <0.05, and employing a two-sided test, we anticipated that the lowest detectable relative risk for stroke would be 1.13.
Data exclusions	We excluded individuals of mothers with missing or incomplete personal identification numbers whose medical records could not be extracted from national patient registers.
Replication	Our findings were derived from register-based data from Denmark and Sweden. They are likely to be generalizable to countries with similar sociocultural contexts and healthcare systems. Replication in other settings would be needed to verify broader reproducibility. The main author has carefully double-checked all steps of the data management and data analyses. We describe in detail the data sources and the applied statistical methods to facilitate reproducibility of the findings in similar Danish-Swedish register data or in other data sources.
Randomization	Our study is an observational cohort study without any randomization.
Blinding	Exposure and outcome data were independently obtained from national register holders, who were unaware of our study. Thus, all group categorizations were based on register data with blinding.

Reporting for specific materials, systems and methods

We require information from authors about some types of materials, experimental systems and methods used in many studies. Here, indicate whether each material, system or method listed is relevant to your study. If you are not sure if a list item applies to your research, read the appropriate section before selecting a response.

Materials & experimental systems

n/a	Involved in the study	
☒	☐	Antibodies
☒	☐	Eukaryotic cell lines
☒	☐	Palaeontology and archaeology
☒	☐	Animals and other organisms
☒	☐	Clinical data
☒	☐	Dual use research of concern
☒	☐	Plants

Methods

n/a	Involved in the study	
☒	☐	ChIP-seq
☒	☐	Flow cytometry
☒	☐	MRI-based neuroimaging

Plants

Seed stocks	*Report on the source of all seed stocks or other plant material used. If applicable, state the seed stock centre and catalogue number. If plant specimens were collected from the field, describe the collection location, date and sampling procedures.*
Novel plant genotypes	*Describe the methods by which all novel plant genotypes were produced. This includes those generated by transgenic approaches, gene editing, chemical/radiation-based mutagenesis and hybridization. For transgenic lines, describe the transformation method, the number of independent lines analyzed and the generation upon which experiments were performed. For gene-edited lines, describe the editor used, the endogenous sequence targeted for editing, the targeting guide RNA sequence (if applicable) and how the editor was applied.*
Authentication	*Describe any authentication procedures for each seed stock used or novel genotype generated. Describe any experiments used to assess the effect of a mutation and, where applicable, how potential secondary effects (e.g. second site T-DNA insertions, mosiacism, off-target gene editing) were examined.*
